# Microwave ablation with a blunt-tip antenna for pulmonary ground-glass nodules: a retrospective, multicenter, case–control study

**DOI:** 10.1007/s11547-023-01672-z

**Published:** 2023-07-17

**Authors:** Zhigang Wei, Jiachang Chi, Pikun Cao, Yong Jin, Xiaoguang Li, Xin Ye

**Affiliations:** 1 Department of Oncology, The First Affiliated Hospital of Shandong First Medical University and Shandong Provincial Qianfoshan Hospital, Shandong Key Laboratory of Rheumatic Disease and Translational Medicine, Shandong Lung Cancer Institute, Jinan, 250014 Shandong China; 2 Cheeloo College of Medicine, Shandong University, Jinan, Shandong China; 3 Department of Interventional Oncology, Renji Hospital, School of Medicine, Shanghai Jiaotong University, 160# Pujian Road, Shanghai, 200127 China; 4 Department of Interventional Therapy, The Second Affiliated Hospital of Soochow University, Suzhou, 215000 China; 5 Department of Minimally Invasive Tumor Therapies Center, Beijing Hospital, National Center of Gerontology, Institute of Geriatric Medicine, Chinese Academy of Medical Sciences, Beijing, 100370 China

**Keywords:** Ground-glass nodules, Microwave ablation, Blunt-tip antenna, Non-small cell lung cancer

## Abstract

**Purpose:**

A previous small-sample study verified that a blunt-tip antenna reduced hemorrhage during microwave ablation. We conducted this large-sample, multicenter, case–control study to further verify the efficacy and safety of microwave ablation with a blunt-tip antenna for ground-glass nodules.

**Materials and methods:**

Patients with pulmonary ground-glass nodules were treated with either a sharp-tip (Group A) or blunt-tip antenna (Group B). A total of 147 and 150 patients were retrospectively allocated to Groups A and Group B, respectively. Group A patients underwent 151 procedures, and Group B patients underwent 153 procedures. We assessed the technical success, technique efficacy, and complications.

**Results:**

Technical success and overall technique efficacy were achieved in all patients (100%). Major complications of pneumothorax were more commonly observed in Group A than in Group B (19.7% vs. 2.0%, *p* < 0.001). Minor complications, such as intrapulmonary hemorrhage (2.0% vs. 9.5%, *p* = 0.005) and hemothorax (0.0% vs. 2.7%, *p* = 0.049), occurred less frequently in Group B compared to Group A.

**Conclusion:**

In the treatment of ground-glass nodules, microwave ablation with a blunt-tip antenna had equal efficacy compared to microwave ablation with a sharp-tip antenna but had a decreased number of hemorrhage and hemothorax complications.

## Introduction

Lung cancer remains the leading cause of malignant tumor-related mortality and morbidity in China [1]. Non-small cell lung cancer (NSCLC) accounts for nearly 85% of all lung cancer cases, wherein adenocarcinoma is the dominant NSCLC type [2, 3]. With the wide application of low-dose computed tomography in cancer screening, an increasing number of patients with ground-glass nodules (GGNs) are identified [4, 5]. GGNs are generally categorized as pure and mixed GGNs and are commonly considered indolent adenocarcinoma subtypes owing to their growth characteristics [6]. Computed tomography (CT) scans every 3–12 months is a reasonable strategy for monitoring GGNs, and the recommended routine follow-up period is up to 5 years (as per the National Comprehensive Cancer Network Clinical Practice Guidelines in Oncology, version 3) [7]. However, even for GGNs that have been stable for 5 years, nodule growth was found in 13.0% of patients with a follow-up of 10 years [8]. Therefore, long-term follow-up and timely intervention are crucial to prevent GGN deterioration.

Currently, lobectomy plus mediastinal lymph node dissection or systematic lymph node sampling is the standard treatment for early-stage (stages I, II, and IIIA) NSCLC. However, lobectomy may not be the preferred choice for patients with GGN-like lung cancer [9, 10]. Patients with GGNs may be histologically diagnosed with adenocarcinoma in situ (AIS) or minimally invasive adenocarcinoma (MIA), and sublobar or segmental resections are the typical treatment regimens [9, 10]. Moreover, a previous study verified that patients with GGNs of 30 mm or less in size and a consolidation-to-tumor ratio of less than 0.5 (in other words, “radiologically noninvasive” GGNs) were not appropriate for lobectomy [11].

Nearly 25% of patients are high-risk for surgery, for whom, stereotactic radiotherapy and image-guided thermal ablation may be alternative treatments [12–15]. Microwave ablation (MWA) has several advantages compared with other thermal ablation methods, such as higher energy, shorter effective time, and less affected by the heat sink effect [16, 17]. Moreover, evidence for MWA as an effective and safe method for GGN-like lung cancer has been shown in several studies [18–21].

As GGNs are usually adjacent to pulmonary vessels or have intertubercular vessels, routine MWA with sharp-tip antennae undoubtedly has a relatively high risk of hemorrhage. Hemoptysis is estimated to occur in 28.8–32.4% of patients [20, 21]. Once hemoptysis occurs, the nodules can be covered and the exact region is difficult to confirm, which may lead to incomplete ablation or even induce the nodules “off target.” To resolve this problem, a new antenna with a blunt-tip was developed [18]. Procedures performed with a blunt-tip may decrease pulmonary vessel damage compared with those performed with a sharp-tip antenna [18]. A previous study explored the safety and efficacy of a blunt-tip and sharp-tip antenna in GGN treatment. It reported similar efficacy for both methods, but blunt-tip antenna was associated with fewer hemorrhage incidences [18]. However, relatively small samples limit the power of these findings; thus, further studies are warranted. Therefore, we conducted this large-sample, multicenter, case–control study to further verify the efficacy and safety of MWA with a blunt-tip antenna for GGNs.

## Materials and methods

### Patients

From January 2018 to December 2021, patients who met the inclusion criteria were allocated sequentially into groups: (1) pulmonary GGNs with a maximum diameter of 30 mm; (2) pathologically verified AIS or MIA; (3) pathologically confirmed invasive adenocarcinoma (IA) unsuitable for radical surgery; (4) pure GGNs or mixed GGNs without histological diagnoses but with no decrease found during follow-up for at least 6 months; (5) GGNs located in the peripheral lung; and (6) adequate cardiopulmonary, hepatic, and renal functions.

The exclusion criteria were as follows: (1) pulmonary lesions larger than 30 mm; (2) pulmonary solid nodules; (3) GGNs identified for less than 6 months; (4) pathologically verified atypical adenomatous hyperplasia or other benign disease; (5) GGNs located in the center of the lung; (6) antiplatelet treatment discontinued for less than 5 days; (7) acute myocardial or acute cerebral infarction during the past 30 days; (8) moderate or serious interstitial pulmonary disease; (9) platelet < 50*10^9/L and abnormal prothrombin/activated partial thromboplastin time. Patients who underwent MWA with the routine sharp-tip antenna or a blunt-tip antenna were assigned to Group A or Group B, respectively.

### Procedures

MWA was performed using CT guidance (LightSpeed 64 VCT, GE Healthcare or uCT 760, United Imaging Healthcare Co., Ltd.). An ECO-100A1 MWA system with either an ECO-100AL6 blunt antenna or ECO-100AI16 sharp antenna (ECO Medical Instruments) or a MTC-3C MWA system with a MTC-3CA-II7 blunt antenna or MTC-3CA-II25 sharp antenna (Vison-China Medical Devices R&D Center, Nanjing, Jiangsu Province, China) was used for MWA. We used a frequency of 2450 ± 50 MHz, and the output power ranged from 0 to 100 W. The commonly applied output powers were 30 W, 40 W, 50 W, and 60 W. For the microwave antenna, the effective length included both 150 mm and 180 mm (two styles) and the dominant outside diameter was 18 G. A water circulation cooling system was employed to cool the surface temperature of the antennae. First, we planned the puncture point and “target skin distance” of the target lesion. Local anesthesia and sedation were achieved with 100 mg of lidocaine and 10 mg of diazepam, respectively. Once satisfactory anesthesia was achieved, MWA was performed by operators from four centers with at least 10 years of experience (Xin Ye, Xiaoguang Li, Yong Jin, and Jiachang Chi have 14, 12, 11, and 10 years of experience, respectively) who inserted the antenna into the target lesion according to the plan. The cold circulating system worked simultaneously during the ablation period. The ablation was terminated when the post-ablation ground-glass opacity surpassed the target lesion by at least 5 mm.

**Fig. 1:**
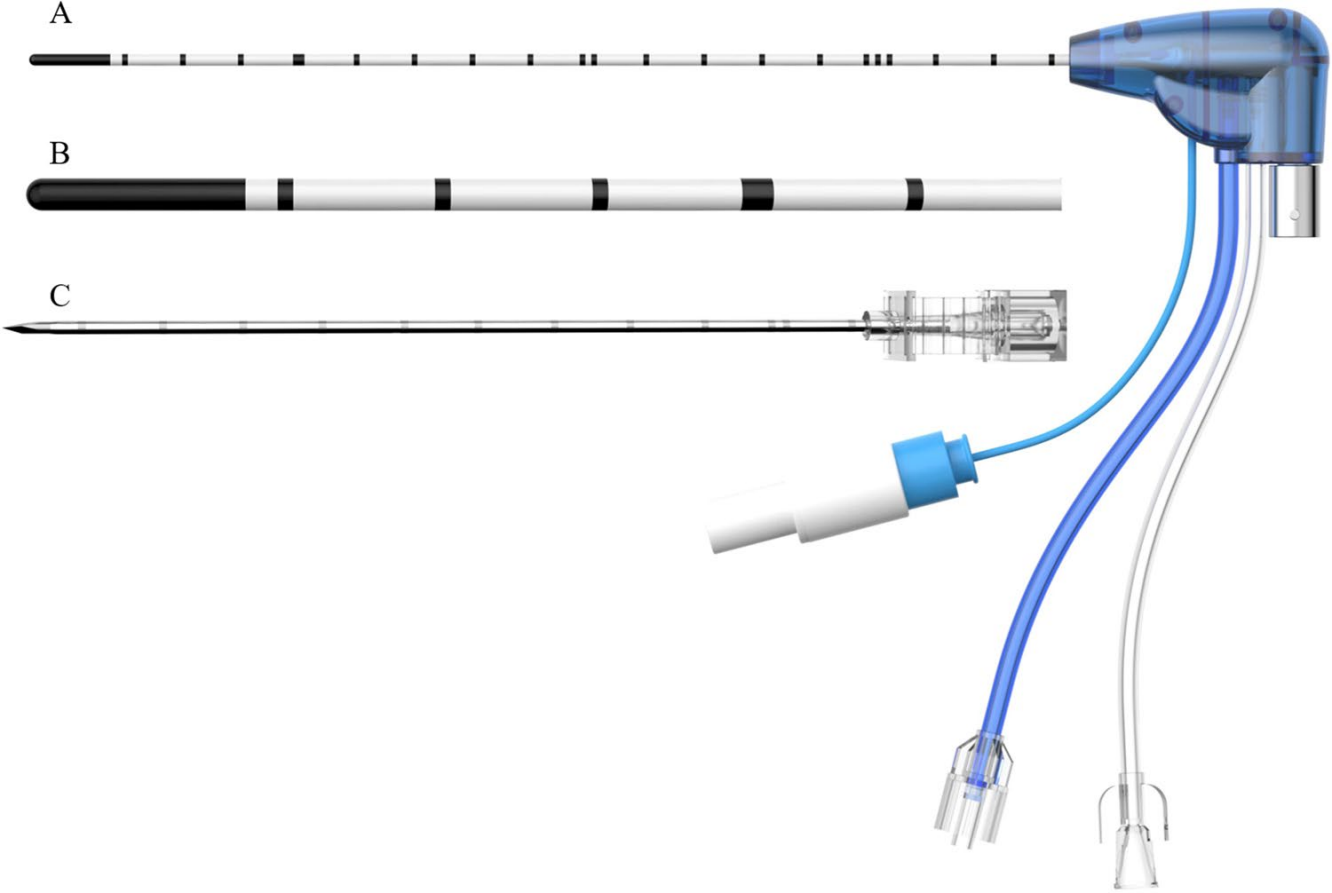
Model of a blunt-tip antenna. **A** Antenna with a blunt-tip; **B** the details of the blunt-tip; **C** a coordinating trocar

For patients who underwent MWA with a blunt-tip antenna (Fig. 1), a coordinating trocar (17 G) was first inserted into the edge of the nodules as planned once anesthesia was achieved. Then, the antenna (18 G) was inserted into the lesion using the trocar. Ablation was performed once the antenna reached the desired position.

### Follow-up

CT was conducted 24 h post-ablation to identify any related complications. Major complications, such as pneumothorax, hemorrhage, and pleural effusion, were treated if necessary. Contrast-enhanced CT (CECT) was conducted 1-month post-ablation and then with an interval of 3–6 months for two years and with 6–12-month intervals for the next 3 years. Thereafter, CECT was performed annually. The last follow up date was April 9, 2022. The follow-up CT images were assessed by both operators and radiologists with at least ten years of experience.

### Response evaluation

Technical success and technique efficacy were used to evaluate the response to MWA. Technical success primarily indicated that the procedure was conducted in accordance with the protocol. In contrast to technical success, technique efficacy rate was the proportion of patients who achieved complete ablation [22]. Complete ablation refers to complete eradication of the known nodules with the following characteristics: (a) the lesion disappears, (b) a cavity forms, (c) fibrosis, (d) solid nodules without contrast-enhanced signs on CT or any fluorodeoxyglucose uptake on positron emission tomography/CT, and (e) lesion in atelectasis without contrast-enhanced signs on CT or any fluorodeoxyglucose uptake on positron emission tomography/CT [23].

### Safety

We assessed MWA complications using the Society of Interventional Radiology (SIR) criteria, which divides all complications into major and minor complications, with grades ranging from SIR A to SIR E. Minor complications were classified as SIR A or B. SIR C and D included major complications. SIR E referred to death, the most serious complication [24].

### Statistical analysis

SPSS software (version 20.0, SPSS Inc.) was used for statistical analyses. Percentage was used to describe categorical variables. Mean and standard deviation/median and interquartile range were used for the description of numerical variables according to the normality test. Two independent samples t-test and nonparametric Mann–Whitney U test were used for the comparison of age and the remaining numerical variables between the groups, respectively. The distribution of baseline characteristics and GGN lesions and incidence of complications between the groups were analyzed using Pearson’s χ^2^ test or Fisher exact test. All statistical analyses were two-sided, and *p* < 0.05 indicated statistical significance.

## Results

### Patient characteristics

A total of 147 and 150 patients were retrospectively allocated to Group A and Group B, respectively. In Group A, the mean age was 58.2 years (range: 29–85 years). Most patients (n = 94, 63.9%) were younger than 65 years of age. Ninety-three patients (63.3%) were women and 122 (83.0%) were nonsmokers. Moreover, 52 patients (35.4%) had comorbidities. Sixty-nine patients (46.9%) were pathologically diagnosed, and IA (37, 25.2%) was the most common histological type.

In Group B, the mean age was 61.2 years (range: 30–87 years). Most patients (n = 85, 56.7%) were younger than 65 years of age. Seventy-six patients (50.7%) were women, and 126 (85.3%) were nonsmokers; 36 (24.0%) had comorbidities. Pathology was performed in 96 (64.0%) patients. AIS, MIA, and IA were confirmed in 36 (24.0%), 33 (22.0%), and 25 (16.7%) patients, respectively. Group A and B differed in baseline characteristics of mean age, sex, comorbidities, and pathology (Table 1).

**Table 1:** Baseline characteristics between the two groups

	Group A (%) (sharp-tip antenna)	Group B (%) (blunt-tip antenna)	*p* value
Number	147	150	
Gender			0.028
Male	54 (36.7)	74 (49.3)	
Female	93 (63.3)	76 (50.7)	
*Age*			
Mean ± SD (range)	58.2 ± 13.0 (29–85)	61.2 ± 12.7 (30–87)	0.042
Age			0.200
< 65	94 (63.9)	85 (56.7)	
≥ 65	53 (36.1)	65 (43.3)	
Smoking history			0.581
Non-smokers	122 (83.0)	128 (85.3)	
Smokers	25 (17.0)	22 (14.7)	
Comorbidity			
No	95 (64.6)	114 (76.0)	0.032
Cardiovascular disease	41 (27.9)	36 (24.0)	0.444
Pulmonary disease	14 (9.5)	0 (0.0)	< 0.001
Type 2 diabetes	19 (12.9)	0 (0.0)	< 0.001
Others	1 (0.7)	0 (0.0)	0.495*
Comorbidity			0.032
No	95 (64.6)	114 (76.0)	
Yes	52 (35.4)	36 (24.0)	
Pathology			< 0.001
No^#^	78 (53.1)	54 (36.0)	
AIS	26 (17.7)	36 (24.0)	
MIA	6 (4.1)	33 (22.0)	
IA	37 (25.2)	27 (18.0)	

*AIS* Adenocarcinoma in situ, *IA* invasive adenocarcinoma, *MIA* minimally invasive adenocarcinoma, *SD* standard deviation

*Fisher exact test. ^#^No, patients with high-risk GGNs and existed during the follow-up of at least 6 months, but not confirmed by pathology

As for the comorbidities, patients of Group A were more common than Group B (35.4% vs. 24.0, *p* = 0.032), which mainly included pulmonary disease (9.5% vs.0.0%, *p* < 0.001) and type 2 diabetes (12.9% vs.0.0%, *p* < 0.001).

### Baseline nodule characteristics

The median maximal diameter was 9.0 mm in both groups, with similar ranges. Most nodules were in the right lung, in the middle and lower lobes. The right lower lobe and the right upper lobe were the most common locations in Groups A and B, respectively (*p* = 0.011). Both the lesion depth (76 mm vs. 77 mm) and the involvement of surrounding pulmonary vessels (91.8% vs. 91.3%) were similar between the two groups. The median power and ablation time in both groups were 40 W and 5 min, respectively (Table 2).

**Table 2:** Baseline characteristics of the ground glass nodules between the two groups

	Group A (%) (sharp-tip antenna)	Group B (%) (blunt-tip antenna)	*p* value
GGN size			0.245*
Median (P25, P75)	9.0 (7.0, 12.0)	9.0 (8.0, 12.0)	0.513
< 10 mm	79 (53.7)	76 (50.7)	
≥ 10 mm, < 20 mm	62 (42.2)	72 (48.0)	
≥ 20 mm	6 (4.1)	2 (1.3)	
GGN location			0.391
Right lung	101 (68.7)	96 (64.0)	
Left lung	46 (31.3)	54 (36.0)	
GGN location			0.065
Upper lobe	55 (37.4)	72 (48.0)	
Middle and lower lobes	92 (62.6)	78 (52.0)	
GGN location			0.011
Right upper lobe	40 (27.2)	40 (26.7)	
Right middle lobe	15 (10.2)	25 (16.7)	
Right lower lobe	46 (31.3)	31 (20.7)	
Left upper lobe	15 (10.2)	32 (21.3)	
Left lower lobe	31 (21.1)	22 (14.7)	
Power of MWA [W, median, (P25, P75)]	40.0 (40.0, 40.0)	40.0 (30.0, 40.0)	0.614
Ablative time [(min, median, (P25, P75)]	5.0 (4.0, 6.0)	5.0 (5.0, 6.0)	0.008
Response to MWA			1.000*
Complete ablation	144 (98.0)	147 (98.0)	
Incomplete ablation	3 (2.0)	3 (2.0)	

*GGN* Ground glass nodule, *MWA* microwave ablation, *P25* percent 25, *P75* percent 75, *W* Watt

*Fisher exact test

### Response and survival

**Fig. 2:**
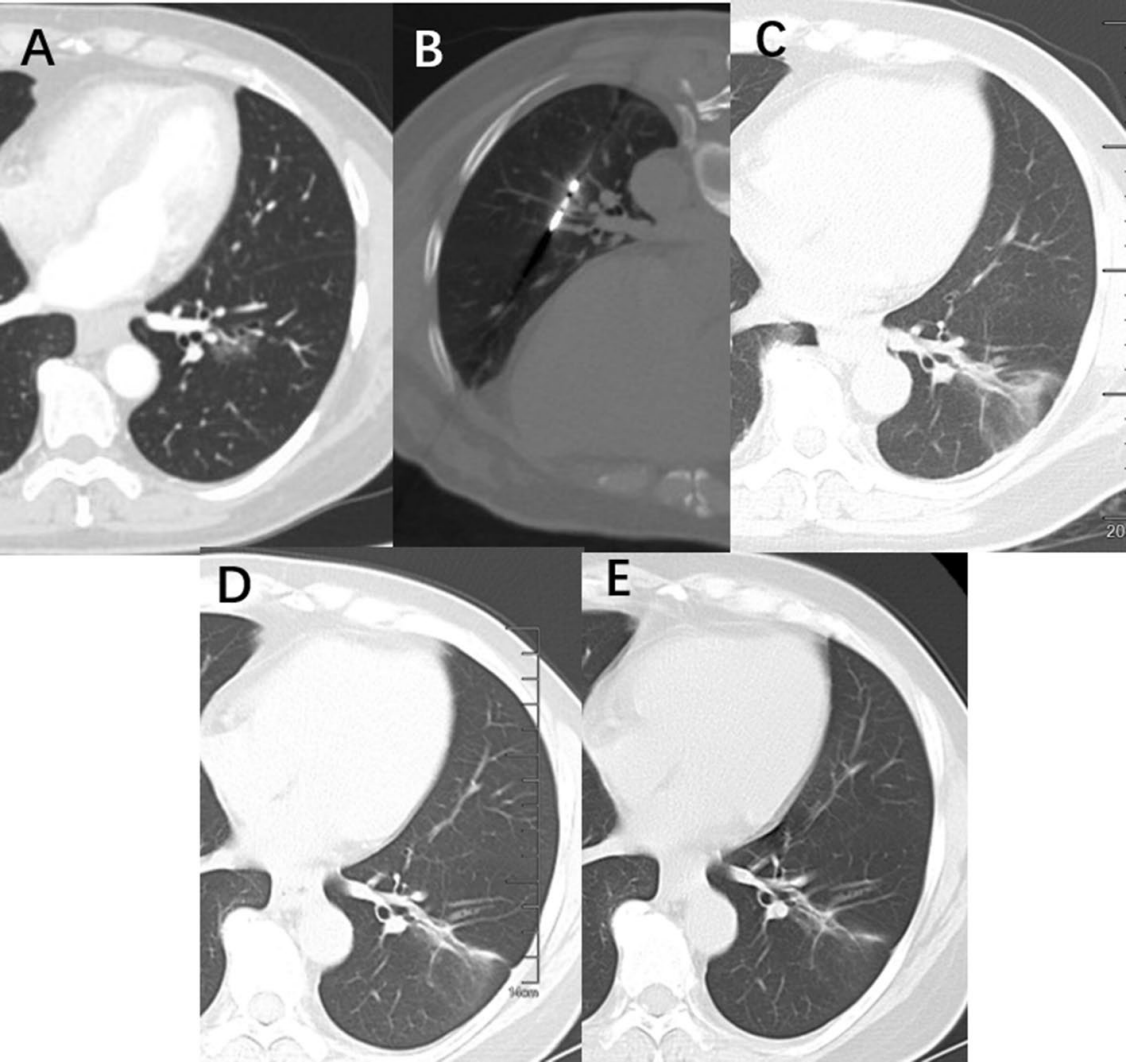
A 48-year-old female patient with pathologically verified MIA underwent ablation with a blunt-tip antenna. **A** A mixed GGN located in the left lower lobe before ablation; **B** a blunt-tip was inserted into the GGN lesion; **C** the GGN lesion decreased 3 months post-ablation; **D** the GGN lesion decreased in advance 12 months post-ablation; **E** the GGN lesion disappeared 24 months post-ablation

**Fig. 3:**
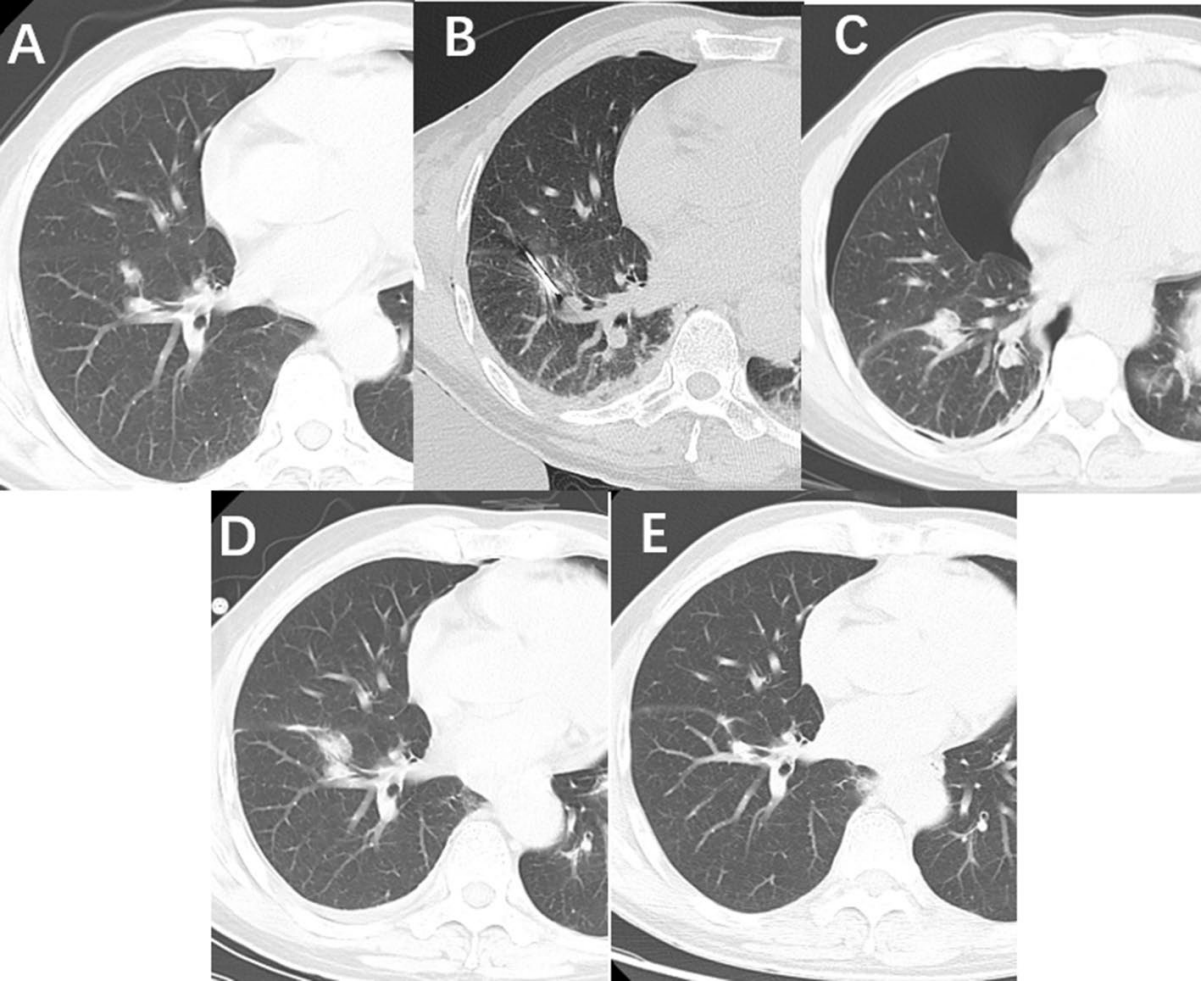
A 58-year-old male patient with pathologically verified MIA underwent ablation with a blunt-tip antenna. **A** A mixed GGN located in the right lower lobe before ablation; **B** a blunt-tip was inserted into the GGN lesion; **C** pneumothorax was observed when ablation procedure was finished; **D** the GGN lesion enlarged 3 months post-ablation; **E** the GGN lesion disappeared 6 months post-ablation

Until April 9, 2022, 151 procedures on 147 Group A patients and 153 procedures on 150 Group B patients (Figs. 2 and 3) were conducted, with a median follow-up of 27.4 and 26.8 months, respectively. Technical success was achieved in all patients (100%). The first technique efficacy reached 98.0% in both groups, and the second technique efficacy was 99.3% and 100% in Groups A and B, respectively. One patient with GGNs located in the right pulmonary apex in Group A underwent three subsequent procedures, and complete ablation was finally achieved.

No patients experienced disease progression and one patient died of cardiovascular disease in Group B during the follow-up period. Neither progression-free survival nor overall survival could be calculated.

### Complications

Regarding complications, no peri-procedure deaths were observed in either group. As well as no major hemorrhagic complications were identified. A minor complication of intrapulmonary hemorrhage was observed in only three patients (2.0%) in Group B, and no interventions were needed; however, 14 patients (9.5%) in Group A experienced intrapulmonary hemorrhage (*p* = 0.005). Moreover, hemothorax was observed in four (2.7%) and zero (0.0%) patients in Groups A and B, respectively (*p* = 0.049). These differences indicate that the blunt-tip antenna decreases the incidence of hemorrhage during ablation. When treated with a blunt-tip antenna, pneumothorax was more commonly identified (28.3% vs. 17.7%, *p* = 0.025) as a minor complication, but fewer patients in Group B than in Group A with pneumothorax as a major complication required chest tube insertion (2.0% vs. 19.7%, *p* < 0.001) (Table 3).

**Table 3:** Incidence of complications between the two groups

Complications	Group A (%) (sharp-tip antenna)	Group B (%) (blunt-tip antenna)	*p* value
*Major complications*			
Pneumothorax	29 (19.7)	3 (2.0)	< 0.001
Pleural effusion	1 (0.7)	1 (0.7)	1.000*
*Minor complications*			
Pneumothorax	26 (17.7)	43 (28.7)	0.025
Intrapulmonary hemorrhage	14 (9.5)	3 (2.0)	0.005
Pleural effusion	4 (2.7)	6 (4.0)	0.750*
Hemothorax	4 (2.7)	0 (0.0)	0.049*

*Fisher exact test

### Discussion

This is the largest multicenter case–control study conducted to date to explore the efficacy and safety of MWA with a blunt-tip antenna and compare it to MWA with a routine sharp-tip antenna. A total of 297 patients were retrospectively enrolled. Technique efficacy was comparable between the groups. The incidence of complications such as intrapulmonary hemorrhage and hemothorax was lower in patients treated with the blunt-tip antenna than in those treated with the sharp-tip antenna.

Patients with GGNs who underwent MWA with a routine sharp-tip antenna have been reported in previous studies [19–21, 25]. Technique efficacy was achieved in all patients (100%) [20, 25]. Only one patient experienced disease progression [20]. In our study, the first technique efficacy was 98.0%, which is lower than that reported in previous studies. We speculate that sample discrimination may have led to this difference. Our study enrolled 147 patients, whereas two previous studies which reported higher first technique efficacy, allocated only 51 and 32 patients, respectively [20, 25].

MWA with a blunt-tip antenna was also reported in one study. Chi et al. [18] compared patients who underwent treatment with a blunt-tip antenna with patients who underwent treatment with a conventional sharp-tip antenna. However, in their study, only 26 patients were included, which is significantly lower than the number of patients included in our study [18]. In our study, although technical success was achieved in patients treated with the blunt-tip antenna and the overall technique efficacy rate of MWA was 100%, three patients failed to achieve complete ablation during the first ablative procedure and underwent a second ablation.

In previous studies, where patients were treated with the conventional sharp-tip antenna, although no major complications of hemorrhage were reported, minor complications of hemorrhage ranged from 19.7 to 28.8% [20, 25]. In instances where patients underwent concurrent biopsy with MWA, the incidence of hemorrhage increased to 32.4–45.0% [19, 21]. The use of a new blunt-tip antenna dramatically decreased the incidence of bleeding [18]. In the study by Chi et al., bleeding in the puncture path and at the lesion were observed in 43.8% and 56.3% of patients treated with a sharp-tip antenna, respectively. Moreover, the corresponding indices in patients treated with a blunt-tip antenna were 15.4% and 0.0% for bleeding in the puncture path and at the lesion, respectively [18]. In our study, blunt-tip antenna application significantly decreased the incidence of both intrapulmonary hemorrhage (2.0% vs. 9.5%, *p* = 0.005) and hemothorax in patients compared to those who underwent a sharp-tip antenna application (0% vs. 2.7%, *p* = 0.049). Nearly all lesions had surrounding pulmonary vessels in both groups. However, the sharp-tip antenna application commonly induced vessel injury and led to bleeding compared with the blunt antenna application.

Similar to our findings, previous studies have reported the occurrence of other complications, mainly pneumothorax, in 9.8–39.0% of patients [19–21, 25] across both groups. We found that the trocar of the blunt-tip antenna, which is triangle shaped, indeed induced more cases of pneumothorax (28.7% vs. 17.7%, *p* = 0.025), but the incidence of major complications of pneumothorax was lower in this group compared to patients treated with the sharp-tip antenna (2.0% vs. 19.7%, *p* < 0.001). Increased incidence at baseline of pulmonary disease in Group A led to more severe cases of pneumothorax than the more common pneumothorax cases. Previous studies have shown that patients with pulmonary disease more commonly experience major complication of pneumothorax, for which chest tube insertion is needed.

Other treatment modalities also serve as alternatives, including the cone-beam CT (CBCT). Compared with CT, CBCT could reduce the time of the thermal ablation procedure in the treatment of lung cancer dramatically [26]; however, the application of CBCT in ground glass opacity is seldom reported.

This study had several limitations. First, this was a retrospective rather than a prospective, randomized study. Second, 53.1% and 36% of patients in both groups did not have histological diagnoses. Thirdly, the median follow-up interval was less than 3 years, which prevented the evaluation of long-term survival. Fourth, different baseline characteristics between the groups may have influenced efficacy and safety. Finally, the patients underwent MWA in four different hospitals and operators, therefore, operator bias may have existed.

In conclusion, in the treatment of GGNs, MWA performed with a blunt-tip antenna had equal efficacy as the use of a sharp-tip antenna, but was associated with fewer complications of hemorrhage and hemothorax.
